# Preparedness for Highly Pathogenic Avian Influenza Pandemic in Africa

**DOI:** 10.3201/eid1310.070400

**Published:** 2007-10

**Authors:** Robert F. Breiman, Abdulsalami Nasidi, Mark A. Katz, M. Kariuki Njenga, John Vertefeuille

**Affiliations:** * US Centers for Disease Control and Prevention, Nairobi, Kenya; † Federal Ministry of Health, Abuja, Nigeria; ‡ US Centers for Disease Control and Prevention, Abuja, Nigeria

**Keywords:** Influenza, avian, Africa, H5N1, capacity, Nigeria, surveillance, pandemic, IDSR, health priorities, research

## Abstract

Global concerns about an impending influenza pandemic escalated when highly pathogenic influenza A subtype H5N1 appeared in Nigeria in January 2006. The potential devastation from emergence of a pandemic strain in Africa has led to a sudden shift of public health focus to pandemic preparedness. Preparedness and control activities must work within the already strained capacity of health infrastructure in Africa to respond to immense existing public health problems. Massive attention and resources directed toward influenza could distort priorities and damage critical public health programs. Responses to concerns about pandemic influenza should strengthen human and veterinary surveillance and laboratory capacity to help address a variety of health threats. Experiences in Asia should provide bases for reassessing strategies for Africa and elsewhere. Fowl depopulation strategies will need to be adapted for Africa. Additionally, the role of avian vaccines should be comprehensively evaluated and clearly defined.

## Abstract 2

Africa’s strategies for pandemic influenza must also strengthen overall public health capacity.

In January 2006, chickens in Kano and Kaduna States in northern Nigeria exhibited diarrhea and respiratory distress and died within a few days. The National Veterinary Research Laboratory in Plateau State isolated influenza viruses from these dying chickens. A Food and Agriculture Organization laboratory in Rome confirmed that the isolates were highly pathogenic influenza A subtype H5N1and genetically similar to influenza (H5N1) clade 2 viruses from China, Indonesia, Japan, South Korea, and other countries (*1*). Official reports of the outbreak among poultry in Nigeria were disseminated on February 7, 2006, which initiated a wide range of bilateral and multilateral responses to the arrival of avian influenza in sub-Saharan Africa. Subsequently, the influenza viruses subtype H5N1 isolated in Nigeria were confirmed as belonging to 3 distinct phylogenetic lineages, which suggested independent introduction into Nigeria through different sources (*2*).

## Response to Influenza (H5N1) in Africa

After highly pathogenic influenza A virus subtype H5N1 was first detected in Africa, plans were announced to implement strategies to eradicate the virus. Nigerian authorities formed a national steering committee led by the ministers of health, agriculture, and information. The committee focused on detection of affected farms, depopulation efforts on those farms and in surrounding areas, surveillance for human disease, and dissemination of messages to promote understanding and modification of behaviors that encourage virus spread. Despite the endorsement of control strategies at the national and state levels, local resources were limited and the virus spread widely. By the end of February 2006, 4 Nigerian states and the Federal Capital Territory, all within central and northern parts of the country, had confirmed influenza virus subtype H5N1 outbreaks in poultry, and presence of the virus was suspected in 9 other states. More than 770,000 birds had either died from illness or were culled. Bilateral donor agencies donated >$3 million, and the World Bank offered $50 million credit to add to several million dollars committed by the government of Nigeria. However, efforts to get human, material, and financial resources rapidly to locally affected areas were inadequate, which stifled well-conceived national control efforts. By early June, 14 Nigerian states had confirmed cases of influenza (H5N1) infection in birds, and several hundred thousand additional birds were reported dead, including chickens, geese, and ducks. Niger and Cameroon had also confirmed influenza (H5N1) outbreaks near the Nigerian border.

Despite continued spread of H5N1 subtype among poultry, human cases of avian influenza were not identified in Nigeria until January 2007, when 1 human case of infection with influenza virus subtype H5N1 was confirmed in a woman. However, weaknesses in existing disease surveillance systems limited capacity to detect transmission of avian influenza to humans. To rule out avian influenza, as of May 2007, specimens from 301 patients have been tested in Nigeria.

That an epizootic of this magnitude in poultry would have negative economic and nutritional effects is not surprising. Nigeria’s estimated 140–160 million poultry account for 10% of its gross domestic product and a substantial proportion of the protein ingested by its 132 million people (*3*). An assessment conducted by the United Nations Development Program showed that the greatest adverse effect was in impoverished areas like rural and semiurban Nigeria, affecting especially backyard and medium-scale farmers*.* Egg and chicken sales declined by >80% within 2 weeks after the announcement of the outbreak; 4 months later, sales were still <50% of baseline. Poultry feed sales also dropped by >80%; 80% of workers on affected farms and 45% on unaffected farms lost their jobs (*4*). The outbreak caused an immediate decline in chicken consumption, even in areas where the disease was not reported among poultry.

In March 2006, avian influenza outbreaks were reported among poultry in Egypt; these reports were followed by 38 laboratory-confirmed cases and 15 human deaths. As of May 2007, influenza virus (H5N1) has been detected in fowl in 8 countries neighboring Nigeria and Egypt (Figure). Outbreaks of avian influenza have continued in Nigeria; as of May 2007, 24 of 36 states had documented avian influenza in poultry. In many other countries in East and West Africa, bird die-offs were reported, but related disease (influenza A subtype H5N1) in humans has not been confirmed. Whether the lack of reported human cases in sub-Saharan Africa, when compared with Egypt, is due to differences in surveillance systems or in animal handling practices that would promote transmission to humans is not clear.

**Figure:**
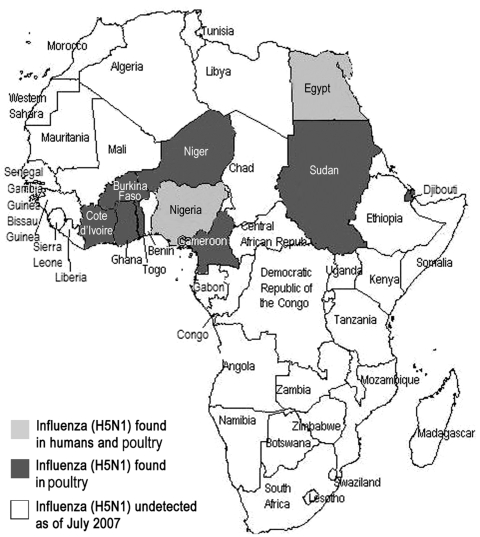
Map of Africa, documenting spread of influenza (H5N1).

## Implications of Avian Influenza Emergence in Africa on Pandemic Preparedness

The next influenza pandemic, if it plays out like the 1918 pandemic, would likely cause more deaths proportionately in sub-Saharan Africa than anywhere else in the world (*5*). Poor nutritional status, high rates of concurrent diseases such as HIV/AIDS, and limited access to healthcare could contribute to high mortality rates (*5*). The devastating effect of pandemic influenza would likely exacerbate unaddressed public health problems and require immense humanitarian emergency assistance, which might not be feasible because of limited available global resources and restrictions on movement of persons worldwide.

Sub-Saharan Africa is uniquely characterized by vast geographic areas that are difficult to access; uneven socioeconomic development; nearly transcontinental limitations in epidemiologic, surveillance, and laboratory capacity; and profound infrastructure weaknesses relating to communications and health systems and capacity of government organizations to effectively focus limited resources. An awareness of this situation has brought substantial pressure on nations in Africa to implement or strengthen detection and rapid response capacities. Animal health strategies have focused on improving surveillance in birds, enhancing laboratory capability to detect influenza A, including H5 strains, and supporting teams to do rapid depopulation. On the human side, an early warning system is needed for an emerging influenza pandemic, i.e., surveillance to detect clusters of influenza-like illness in healthcare facilities and communities and to rapidly confirm them as influenza in the field and at qualified laboratories.

Effective preparedness and responses will require fresh, innovative thinking relevant to local circumstances, determination, political will, and national and international resources. The African Regional Office (AFRO) of the World Health Organization (WHO) and its country offices have a clear mandate to address regional impending and ongoing health crises. Partners, including other United Nations agencies, development organizations, and allied nongovernment organizations, should line up with AFRO and national ministries of health and livestock to encourage and catalyze multisectoral government and nongovernment commitment and action.

## Balancing Pandemic Influenza Concerns with Ongoing Health Priorities

The potential effects of pandemic influenza on human health and political stability in sub-Saharan Africa cannot be denied. Of paramount concern, however, is that this potential disaster emerges onto a backdrop of countries struggling to address epidemics of HIV, tuberculosis, and malaria; resurgence of paralytic poliomyelitis; and high childhood mortality rates due to pneumonia and diarrheal diseases. Many of the severe effects of these diseases could be prevented by use of proven public health tools (e.g., antiretroviral drugs; improved detection and treatment of tuberculosis; insecticide-treated bednets; vaccines against *Streptococcus pneumoniae*, *Haemophilus influenzae* type b, and rotavirus), but African countries lack the resources and infrastructure to take full advantage of these tools.

In fact, the frenetic global activity around avian influenza’s potential threat to human health is diverting critical financial and human resources and focus from the real ongoing, distressing effects of the major infectious disease syndromes. This diversion may be acceptable for a short time if the most pressing challenges for preparedness can be quickly and effectively addressed. However, for many months, ministries of health personnel in countries throughout Africa have been deeply immersed in pandemic preparedness and response planning; they have been pulled away from routine activities, and critical programs have been put on hold. In Nigeria, while senior health officials were struggling to maintain Global Fund resources for HIV and malaria, as well as to improve polio vaccination coverage and stop the export of polio to other nations, considerable immediate pressure was exerted to ramp up influenza surveillance, containment, and preparedness activities after the initial confirmation of influenza virus (H5N1). Similarly, when avian influenza was confirmed in poultry in Juba, Sudan, in September 2006, the newly formed Ministry of Health staff of the government of South Sudan had to balance already demanding commitments to battle HIV, tuberculosis, and an ongoing cholera epidemic with the need to prepare for potential influenza A virus (H5N1) infection of humans. The substantial effort required added more weight to a grossly overstressed public health system.

Tension between existing disease concerns and the potential threat of a pandemic raises several questions: whose concern is avian influenza; and what are appropriate, balanced responses, particularly in countries with severely limited public health resources and overwhelming health problems? A pandemic strain emerging in Africa is a global concern as much as an African concern. Without rapid detection systems and effective control measures, a highly transmittable strain would quickly spread globally. Wealthier nations have recognized this and pledged nearly $2.5 billion to address the problem, although most is not specifically for use in Africa.

In addition, an influx of massive resources from more developed nations, if not well coordinated and balanced in scope, could distort the sense of priorities and damage other critical public health and agricultural programs. How then can a balance be achieved and result in appropriate enhancements to address the threat of pandemic influenza while keeping focus on the existing major health priorities facing Africa? One approach, detailed below, would be to use new resources in a way that is broadly applicable to building public health capacity in recipient nations.

## Improving Pandemic Preparedness

The need and resources for rapid detection of potentially pandemic strains of influenza should be leveraged with support for surveillance (Table) for other epidemic-prone and vaccine-preventable diseases; parallel, vertical systems will ultimately not be sustainable in Africa. The drive for improved influenza surveillance can fuel the implementation of Integrated Disease Surveillance and Response (IDSR), promoted by WHO and its partners and endorsed in 1998 by 46 countries in the African region (*6*–*8*). IDSR includes surveillance for vaccine-preventable diseases and epidemic-prone diseases such as meningococcal disease, measles, cholera, typhoid, yellow fever, and viral hemorrhagic fever. If functioning well, IDSR should provide health officials with signals when disease incidence passes thresholds, suggesting an impending epidemic and the need to ramp up disease prevention activities like vaccination, safe water campaigns, or quarantine. Presently, however, IDSR is functional in a few countries, including Eritrea, Ghana, and Kenya. Strengthening IDSR throughout Africa would create an effective early warning system capable of detecting a pandemic strain of influenza or clusters of patients with severe acute respiratory illness, which could signal the beginning of person-to-person transmission. Early detection of a pandemic would facilitate the timely implementation of pharmaceutical and nonpharmaceutical containment measures. In addition, strengthening IDSR would have the dual benefit of enhancing capacity for early detection of outbreaks of more conventional diseases and perhaps currently unrecognized threats.

**Table:** Surveillance enhancements needed to address pandemic influenza and other emerging threats, Africa

Method	Needs
Integrated disease surveillance and response	Resources, training, local commitment
Surveillance among health workers, students	Standard operating procedures, resources
Mobile phone messaging	Resources and targeted application of existing technologies
Veterinary surveillance for zoonoses	Political will to encourage stronger linkages between health and animal ministries, resources, effective models to emulate

For IDSR to effectively function as an early warning system for influenza and other communicable diseases, a number of steps should be taken. First, additional resources should be made available for field training of national and local staff and subsequent site assessments. Second, this conventional public healthcare facility–based surveillance system should be expanded to cover private healthcare institutions and include community-level surveillance, through reporting by village chiefs, traditional healers, dispensaries, and primary schools. Finally, existing technology in Africa should be leveraged to make IDSR a user-friendly, more efficient surveillance system. The widespread use of mobile telephones and extensive mobile phone networks throughout Africa could be extremely helpful for reporting unusual health occurrences through inexpensive, potentially automated, text messaging to an identified, widely recognized, and toll-free number. Reports of disease clusters could then be immediately verified and investigated by public health personnel (*9*). This tool could also be used for animal surveillance.

## Intersectoral Communication and Collaboration

With similar urgency, enhanced veterinary surveillance and strong, functional networks of communication and collaboration between animal and human health experts would increase potential for prevention of transmission of influenza from animals to humans. Currently, linkages and communication mechanisms between health and veterinary officials are neither explicit nor functional. A major challenge in Nigeria’s response to avian influenza (H5N1) was to ensure communication between Ministry of Health and Ministry of Agriculture staffs on the ground so that when poultry outbreaks occurred on farms, the Ministry of Health was alerted to look for human cases.

## Laboratory Capacity

Resources for improving infrastructure to address threats of avian influenza must be channeled to improve local laboratory capacity and strengthen laboratory networks. WHO and AFRO have a critical role to promote, select, develop, train, and sustain laboratory capacity for diagnosis of influenza within regional reference laboratories and national influenza centers. AFRO has already designated several existing laboratories in Africa as regional reference laboratories for testing seasonal and avian influenza in humans. With enhanced surveillance, these laboratories should be able to rapidly confirm or rule out influenza as the cause of a cluster of severe respiratory illness, which would allow for rapid epidemic responses. Already, laboratories in several countries in Africa have been upgraded, laboratorians have been trained, equipment (real-time and conventional PCR machines) and appropriate reagents (primers and probes) have been procured, and quality control mechanisms have been put in place so that PCR can be used in a timely manner to reliably document the presence of highly pathogenic influenza A virus subtype H5N1. With additional reagents and training, these laboratories could have the capacity to rapidly detect causes for other outbreaks and assist with surveillance for routine and emerging diseases. As laboratory surveillance is initiated and strengthened, training, leveraging of equipment maintenance and service contracts, and resource sharing across animal and human facilities will be essential.

## Epidemiologic Capacity

Being prepared for an influenza pandemic will require having a well-trained corps of epidemiologists. Field epidemiology training programs and Public Health Schools without Walls are in place in several countries in Africa; these programs are successfully training medical doctors (and laboratorians in the case of Kenya’s Field Epidemiology and Laboratory Training Program) to become public health experts. To minimize out-migration, graduates should be rewarded through career advancement pathways within national public health systems.

## Response and Containment

In a joint effort by WHO and the Centers for Disease Control and Prevention, rapid outbreak response teams are being trained in sub-Saharan African countries to recognize outbreaks, collect information and specimens, and implement quarantine measures, if indicated, while using and distributing personal protective equipment and antiviral drugs. Training rapid response teams in each country is a relatively new concept for Africa, but such teams in Asia have responded to dengue epidemics and, more recently, to avian influenza. If these teams are functional, they will strengthen responses in Africa to a wide array of epidemics, enabling timely implementation of interventions to prevent illness and death.

Should a major health disaster such as an influenza pandemic occur, essentially no capacity exists to appropriately address the healthcare crisis while continuing to deal with other routine health problems, which themselves can at times overwhelmingly stress healthcare systems. Preparation for a pandemic must strengthen hospital capacity to optimally and safely manage severely ill patients with potentially highly contagious illnesses. Although aiming for parity with hospitals in industrialized nations on quality standards for infection control is excessively ambitious, simple, practical approaches to implement basic infection control measures developed by WHO can be used (*10*).

Although data on the topic are limited, public awareness of avian influenza is minimal in Africa, specifically concerning the link between dying birds and human illness and the potential of catastrophic emergence of pandemic influenza. The United Nations Children’s Fund has conducted participatory action research, which has shown that while community outreach campaigns will be needed across the continent, messages must be locally customized because of wide variations in literacy, awareness, experience, and beliefs (*11*). Recent efforts to strengthen information, education, and communication in Africa will improve societal mobilization for behavior modification capacity, which can be called upon to address public health threats (*12*). Ultimately, by developing effective health communication measures, functional surveillance systems with strong epidemiologic and laboratory support, and capable multisectoral rapid response teams, it may be possible to curtail spread of a lethal pandemic strain and buy time to develop and deliver effective human-administered vaccines (*13*,*14*).

## A Call for New Strategies

In Asia, the principal control approach has been depopulation of poultry in affected and nearby farms along with attempts to restrict movement of poultry and poultry products from affected areas. Even when aggressively pursued, these efforts were not always timely or comprehensive, and avian influenza became endemic among birds in many parts of Asia and has now spread to Europe and Africa. The diffuse spread of the virus in avian species and some transmission to humans and other mammals has provided numerous opportunities for a pandemic virus to appear; however, a strain easily transmittable from person to person has not yet emerged. Although this provides some comfort for now, a variety of ecologic and health factors could affect the emergence of a devastating pandemic strain.

The massive geographic spread of avian influenza in birds should cause a re-evaluation of bird depopulation as the principal strategy for control. Although depopulation appears to have worked when the virus was localized during 1997 in Hong Kong and 1.5 million birds were culled, widespread presence of the virus in migratory and domestic birds has severely limited the usefulness of this approach. In addition to direct costs, massive culling operations dramatically affect the economic and nutritional status of impoverished people. Compensation programs, critical to success of culling efforts, have not been implemented optimally in most countries where influenza virus (H5N1) epizootics have occurred. Without credible, adequate, and rapidly implemented compensation programs linked to depopulation efforts, farmers are not encouraged to report bird die-offs and may transport birds to unaffected areas, thereby spreading the virus (*15*). Furthermore, depopulation may counter evolutionary pressure. If the birds that survive local outbreaks are the fittest, killing them provides no evolutionary advantage to survival.

Vaccination of poultry may be a useful adjunct to depopulation in a defined area surrounding an affected farm (ring depopulation). Some available vaccines seem to provide immunity to infection without substantially prolonging viral shedding (*16*–*19*). If so, focusing vaccination programs on farms around affected areas may be a less costly, more socially acceptable, and more effective solution than mass culling, with or without compensation. Effective use of influenza vaccines in avian populations could theoretically reduce the risk for emergence of a pandemic strain by decreasing the numbers of circulating avian influenza strains that could mutate or reassort with other circulating influenza viruses (*19*). Additionally, use of emergency vaccination can be effective in conjunction with other measures at the time of poultry outbreaks (*19*). Ultimately, control measures will be implemented by agriculture and livestock ministries, which must address potentially conflicting commercial and public health interests.

Current vaccine supplies for poultry in risk areas may be insufficient. A clear avian vaccination strategy, articulated and implemented by international organizations, would encourage adequate production of effective vaccines for use in currently and prospectively affected areas. Analyses comparing costs and potential cost-effectiveness of depopulation and vaccination strategies (and strategies that use both approaches) would be helpful.

## The Path Forward

Activities to address the threat of pandemic influenza should, whenever possible, build upon existing public health systems and enhance available health infrastructure rather than create new systems. In Vietnam, for instance, research organizations like Oxford University and The Wellcome-Trust, and partners they support, were able to refocus their efforts onto avian influenza and provide substantial new knowledge relevant for protecting health (*20*,*21*). Likewise in Africa, many nongovernment and religious organizations can build on their existing networks and groundwork to assist governments with surveillance and risk-reduction efforts, as has been the case for initiatives to address HIV/AIDS, provide safe water, and eradicate poliomyelitis (*22*–*26*).

Identifying existing laboratory and disease-reporting mechanisms in individual countries is crucial so they can be augmented to achieve sustainable capacity for surveillance for influenza and other emerging diseases. National disease reporting systems—IDSR and polio surveillance—may provide entry points for such activity but will require strategic investments that in large part need to be defined in the context of national priorities and balanced with global needs. Early detection and improvement of the continent’s preparedness for emerging diseases has been brought into focus by the recent adoption of a treaty by the World Health Assembly of new International Health Regulations (IHR [2005]), which now compels countries to have more effective surveillance and reporting systems to improve the ability for early detection of global disease threats (*27*). More specific to influenza, IHR (2005) compels countries to implement optimal communications, rapid risk assessment, and containment and response measures, should a pandemic strain emerge.

An old expression says, “You can’t slide uphill.” The difficult work associated with preparedness for pandemic influenza will help bring Africa closer to the top of the hill, making it possible to efficiently respond to this and other threats and, when necessary, slide toward a beneficial outcome.
